# Nipple shield use in preterm infants: Prevalence, motives for use and association with exclusive breastfeeding—Results from a national cohort study

**DOI:** 10.1371/journal.pone.0222811

**Published:** 2019-09-20

**Authors:** Ragnhild Maastrup, Sisse Walloee, Hanne Kronborg

**Affiliations:** 1 Knowledge Centre for Breastfeeding Infants with Special Needs, Department of Neonatology, Rigshospitalet, Copenhagen University Hospital, Copenhagen, Denmark; 2 Research Unit Women’s and Children’s Health, Juliane Marie Centre, Rigshospitalet, Copenhagen University Hospital, Copenhagen, Denmark; 3 Department of Obstetrics and Gynecology, Slagelse Hospital, Slagelse, Denmark; 4 Department of Public Health, Section for Nursing, Aarhus University, Aarhus, Denmark; University of North Carolina at Greensboro Center for Women's Health and Wellness, UNITED STATES

## Abstract

**Background and aim:**

Prevalence and motives for nipple shield use are not well studied in preterm infants and recommendations of nipple shield use in preterm infants are inconsistent. The aim of this study was to determine the prevalence of nipple shield use, explore the motives for nipple shield use and elucidate the association with exclusive breastfeeding in preterm infants.

**Methods:**

The study was part of a prospective survey of a Danish national cohort of preterm infants based on questionnaires answered by the 1221 mothers of 1488 preterm infants with gestational age of 24–36 weeks. Data on nipple shield use was available for 1407 infants.

**Results:**

Nipple shields were used by 54% of the mother-infant dyads for many different motives and was more often related to breastfeeding problems associated with the infant than with the mother. The most common motive for nipple shield use was “infant slipped the nipple” (52%). The lower the gestational age, the more frequently nipple shields were used for motives related to the infant. For those using a nipple shield, only the motive “infant fell asleep at the breast” was associated with a higher risk of not breastfeeding exclusively at discharge (OR 1.90 (95% CI 1.15; 3.13), p = 0.012), and “breast too engorged” with a lower risk of not breastfeeding exclusively (OR 0.32 (0.16; 0.63), p = 0.001), but overall nipple shield use was associated with failure of exclusive breastfeeding.

**Conclusion:**

The present study does not give justifiable motives for nipple shield use, except for “breast too engorged”. Nipple shields should not be recommended for infants falling asleep at the breast, instead, staff and mothers should be patient, allowing the dyad time skin-to-skin. The results indicate that the use of a nipple shield does not promote exclusive breastfeeding in preterm infants.

## Introduction

The prevalence of nipple shield use in preterm infants differs between studies. In a UK study, 2% of 562 preterm infants used a nipple shield [1]; in two Danish studies 54% of 1488 and 41% of 373 preterm infants used a nipple shield [2, 3]. High prevalence of nipple shield use are anecdotally reported in the US after two US studies of 15 and 34 preterm infants in 1996 and 2000 found that preterm infants consumed more milk with a nipple shield than without and recommended the use for breastfeeding establishment in preterm infants [4, 5]. Reported motives for nipple shield use in preterm infants from studies including 12 and 34 nipple shield users were flat or inverted nipples, accustomed to a firmer teat, poor latch, infant falling asleep soon after being positioned at the breast, and maternal nipple discomfort [1, 5]. In a survey of health care professionals’ attitudes, the most common motive for recommending use of a nipple shield was to help preterm infants born before 35 gestational weeks latch [6].

Few have investigated the association between nipple shield use and breastfeeding duration. Studies of full-term infants as well as preterm infants have found negative associations between early use of nipple shield and exclusive breastfeeding duration [2, 3, 7]. A national cohort study of 1488 preterm infants showed that those who used a nipple shield had a 2.3 higher OR for not being exclusively breastfed at discharge from NICU to home, and they did not establish breastfeeding earlier than the 46% not using a nipple shield [2, 8]. The US study including 34 preterm infants concluded that nipple shield use was not associated with decreased duration of any breastmilk feeding [5].

The use of a nipple shield is not recommended for full-term infants [9] because of the negative impact on exclusive and any breastfeeding duration [3, 10], and less optimal weight gain [7]. For preterm infants the recommendations are inconsistent. The evidence-based expansion of the Baby-friendly Hospital Initiative for neonatal wards (Neo-BFHI) has set a standard that “Nipple shields should not be used routinely in the neonatal ward. They should only be used after the mother has received skilled support in solving the underlying breastfeeding problem, and after the mother’s repeated attempts to breastfeed her infant without the shield” [11]. Some hospitals recommend nipple shield use in preterm infants as a standard of care [12], and others recommend that every infant who shows sign of immature suction pressure could potentially benefit from using a nipple shield [13]. To inform practice we need further evidence-based knowledge about the early use of nipple shield among preterm infants based on studies including more participants: How often is a nipple shield used and by whom? Are there differences in nipple shield use by gestational age (GA) groups? When is nipple shield use initiated and why and how do the motives for nipple shield use relate to exclusive breastfeeding? This study intends to answer the questions based on results from the national cohort of 1488 preterm infants (2).

### Aim

The aim of the present study was first to determine the prevalence of nipple shield use among preterm infants and to explore the motives of nipple shield use and elucidate the association with exclusive breastfeeding in preterm infants. The aim was achieved.

## Materials and methods

### Ethics statement

This study was conducted in accordance with the Declaration of Helsinki [14] and approved by the Danish Data Protection Agency (j.nr. 2009-41-4024). The survey did not, according to the Danish law, need approval by the Biomedical Research Ethics Committee. Written consent to participate was obtained from mothers of the preterm infants.

### Design

The study was part of a prospective survey of a national Danish cohort of preterm infants which used questionnaires and structured telephone interviews conducted from September 2009 to December 2011. This article is the third from the cohort [2, 8].

### Setting

Denmark has 5.5 million inhabitants and about 60,000 births per year, 7% of which are premature (born before 37 gestational weeks). Public health care is provided to all citizens, with equal access to health care in public hospitals free of charge [15]. Denmark has 19 NICUs of which four provide high-intensive care, 14 medium-intensive care, and one low-intensive care. Preterm infants born before 35+0 gestational weeks are, in general, admitted to one of the NICUs; those born between 35+0 and 36+6 are only admitted if they need observation or treatment [16].

In general, Danish preterm infants are hospitalised until breastfeeding is well established or until exclusive breastfeeding is no longer the goal and mixed feeding or bottle-feeding established [16]. Bottle-feeding is not introduced to preterm infants when exclusive breastfeeding is still the mother’s goal. Mothers have unrestricted access to their infants in 90% of the NICUs [16]. Former analyses of this cohort found that 29% of the mothers were admitted to the same ward as their infant during the whole hospital stay, and daily skin-to-skin contact was common as soon as the infant tolerated transfer between parent and incubator [2, 8]. Whether to use nipple shield or not would mostly be decided by the staff who helps the mothers with breastfeeding.

### Participants

All preterm infants who were admitted to the participating NICUs from 1 September 2009 to 31 August 2010, were eligible. Exclusion criteria were infant discharge to maternity units before five days of age, interpreter not available for a non-Danish speaking mother or neonatal death. Mothers who did not plan to breastfeed and did not initiate breastmilk expression participated only with the first questionnaire, and their data were not included in the analyses in the present paper.

All wards in Denmark that routinely take care of preterm infants during breastfeeding establishment participated in the study, which included 18 of the 19 NICUs, two special care units and one children’s department; 18 of the 21 participating units adhered to the project protocol [2]. The data set used in the present paper contains personal identifiable data and is by Danish law not publicly available.

### Instruments and data collection

Based on a review of the literature and a national expert panel, three study-specific questionnaires for mothers of preterm infants were developed to explore the use of various clinical practises to facilitate breastfeeding. The national expert panel consisted of eight neonatal nurses with experience in breastfeeding of preterm infants and research and four of them were International Board Certified Lactation Consultants.

The questionnaires included demographic questions about mother and infant, the infant’s breastfeeding progression and clinical practice. Questionnaire 1 and 2 consisted of 38 and 59 questions, respectively, and were filled out by the mother approximately one week after delivery and at the infant’s discharge from NICU to home. Questionnaire 3, with 17 questions, was used for structured telephone interviews conducted by nurses from each ward with breastfeeding mothers at the infant’s one, four, six, and 12 months corrected age or until breastfeeding ceased, whichever occurred first. More details and the questionnaires can be found in a previously published paper [2].

### Study and outcome variables

The primary study variables included nipple shield use in mother-infant dyads, date for initiation, motives for nipple shield use and demographic variables. The mother was asked to tick off motives for initiating use of a nipple shield. Even if the use of nipple shield was not the mother’s idea, she would explain the staff´s motive for suggesting a nipple shield. Since she could choose as many motives as relevant for her, adding all motives in the table will give more than 100%. She was not asked which motive was the most important.

Main outcome variable in the present study was exclusive breastfeeding at discharge from NICU to home. Secondary outcome variable was exclusively breastfeeding at one month corrected age.

Whether nipple shield use affects breastfeeding duration could depend on the definitions of breastfeeding. Because we wanted to know if nipple shield use affected the infant’s ability to continue sucking at the breast and extract milk from the breast, exclusive breastfeeding was defined as feeding at and from the breast, not including feeding breastmilk from a bottle [17].

### Definitions of terminology

- Exclusive breastfeeding was defined as an infant feeding directly at and from the breast and could include medication and vitamins.
- Partial breastfeeding includes other feeding methods (such as bottle, cup, lact-aid, regardless of content) in addition to direct breastfeeding.
- Any breastfeeding includes exclusive and partial breastfeeding.

Age definitions [18]:

- Chronological age = postnatal age: time elapsed from birth
- Postmenstrual age: GA plus chronological age
- Corrected age: chronological age reduced by the number of weeks born before 40 weeks of gestation

Preterm infants were divided into four groups depending on GA in weeks + days [19, 20]:

- Extremely preterm infants: GA 24+0–27+6
- Very preterm infants: GA 28+0–31+6
- Moderate preterm infants: GA 32+0–34+6
- Late preterm infants: GA 35+0–36+6

For this study, the lower limit for late preterm infants was set at 35+0 weeks and days, given that preterm infants in Denmark with GA less than 35+0 weeks and days, regardless of their physical situation, are admitted routinely to a NICU.

### Statistical analyses

SPSS version 22.0 was used for statistical analyses. Descriptive statistics were used to present mother and infant characteristics. The normally distributed results are reported with mean and standard deviation (SD); the remaining results are reported with median, interquartile range (IQR) or percentages [21]. Pearson’s Chi-Square test was used to determine statistically significant differences between those using and not using nipple shields. Nipple shield use was analysed in all infants, in GA groups, and in the participating wards. Spearman’s rank correlation test was used to determine statistically significant correlation between NICUs with rates of nipple shield use and low rates of exclusive breastfeeding. A scatter plot was used to illustrate correlation between GA and postmenstrual age at initiation of nipple shield use, and between GA and days from first breastfeeding attempt to nipple shield initiation. Breastfeeding duration for infants lost to follow up were adjusted to the time at the latest answered questionnaire and the analyses performed with all infants. A multiple regression analysis was performed with mothers self-reported motives for nipple shield use to explore any associations with exclusive breastfeeding at discharge and was adjusted for variables known to be associated with exclusive breastfeeding in preterm infants at discharge [2]. The multiple regression analysis was performed with one infant per mother to ensure that mothers of twins did not count as double [21] (for multiple births, the first-born infant was included if both/all used a nipple shield). Values of p <0.05 were considered statistically significant.

## Results

**Fig 1:**
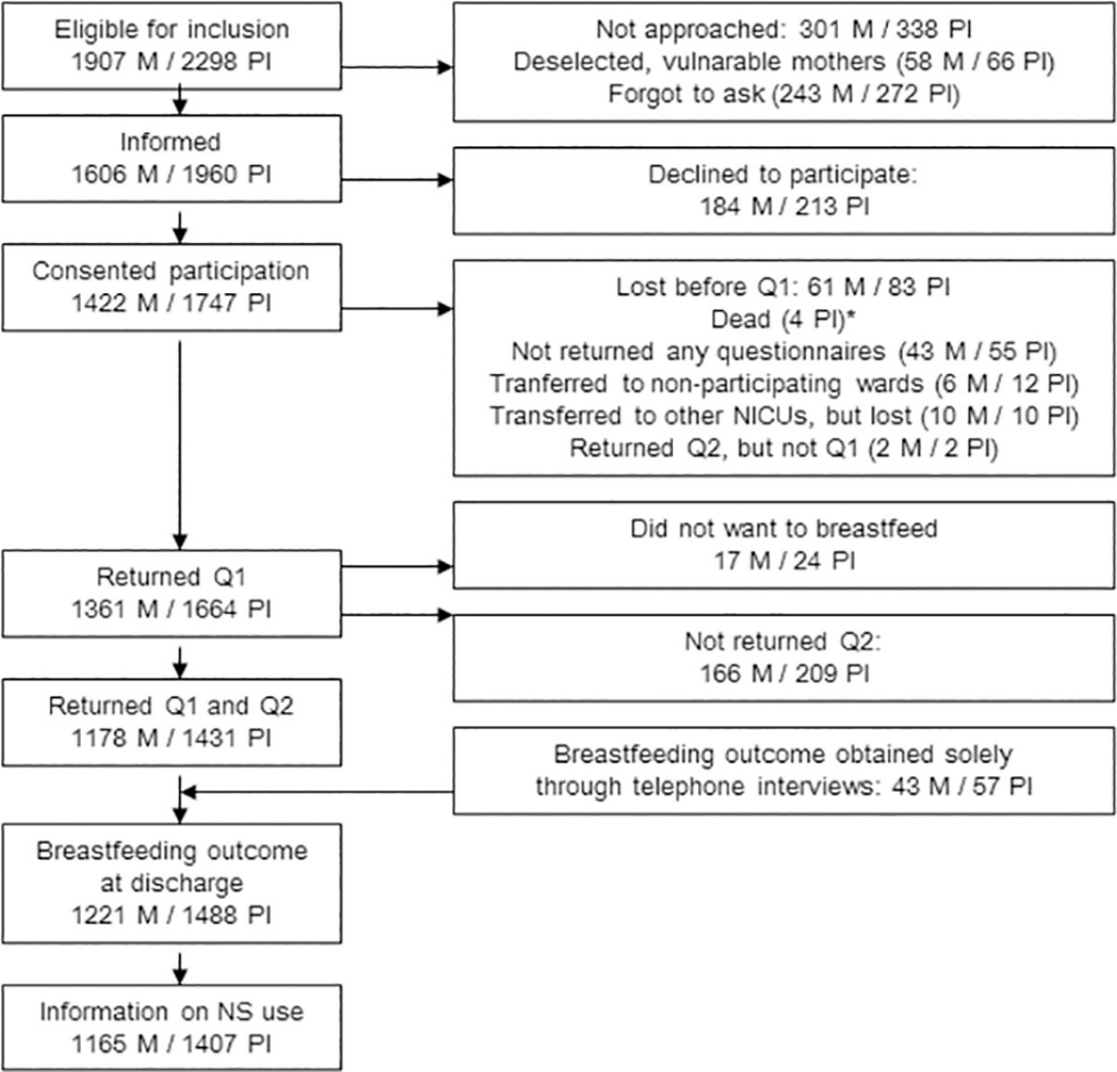
Flowchart. M = mothers, NICUs = Neonatal Intensive Care Units, NS = nipple shield, PI = preterm infants, Q1 = questionnaire 1, Q2 = questionnaire 2.

Data on nipple shield use was available for 1407 preterm infants (1165 mothers) from a national cohort of 1488 preterm infants whose 1221 mothers planned to breastfeed, see Flowchart in Fig 1.

### Prevalence, characteristics and initiation of nipple shield use

Nipple shields were used by 54% of mother-infant dyads. Nipple shields were used significantly more by mothers of infants small for gestational age, first-time mothers, mothers younger than 30 years. Nipple shields were used less by mothers with high breastfeeding self-efficacy, mothers for whom breastfeeding was of very great importance, smoking mothers, and mothers speaking another language than Scandinavian at home. More mothers who used nipple shields during the NICU stay continued breast milk pumping at discharge (Table 1).

**Table 1:** Infant and mother characteristics in nipple shield users and non-users.

Infant characteristics (1407 infants)	Total n/N	%	Used nipple shield (%)	Not used nipple shield (%)	Person’s Chi -Square
**GA <28 weeks**	**54/1407**	**4**	4	3	0.332
**GA 28–31 weeks**	**237/1407**	**17**	18	16	0.253
**GA 32–34 weeks**	**647/1407**	**46**	47	45	0.629
**GA 35–36 weeks**	**469/1407**	**33**	31	36	0.061
**Multiples**	**487/1407**	**35**	35	35	1.000
**SGA**	**253/1393**	**18**	21	15	0.003
**Maternal characteristics (1165 mothers)**					
**Breastfed excl for at least 4 months**	**185/1119**	**17**	9	27	<0.0001
**Primiparous**	**732/1153**	**64**	77	47	<0.0001
**Very confident in fulfilling plans of breastfeeding duration**	**291/1144**	**25**	21	31	0.0003
**Breastfeeding is of very great importance to the mother**	**678/1148**	**59**	54	65	0.0001
**Maternal age < 30 years**	**490/1164**	**42**	46	38	0.009
**Maternal education, low**	**385/1151**	**33**	33	35	0.469
**Intermediate**	**543/1151**	**47**	47	47	0.839
**High**	**223/1151**	**19**	20	19	0.544
**Speaking another language than Scandinavian at home**	**78/1160**	**7**	5	9	0.011
**Smoking**	**116/1153**	**10**	8	12	0.035
**Delivery mode caecarean section**	**586/1163**	**50**	52	49	0.316
**Continuing breast milk pumping at discharge**	**529/1158**	**46**	55	34	<0.0001
**GA = gestational age**					

**Fig 2:**
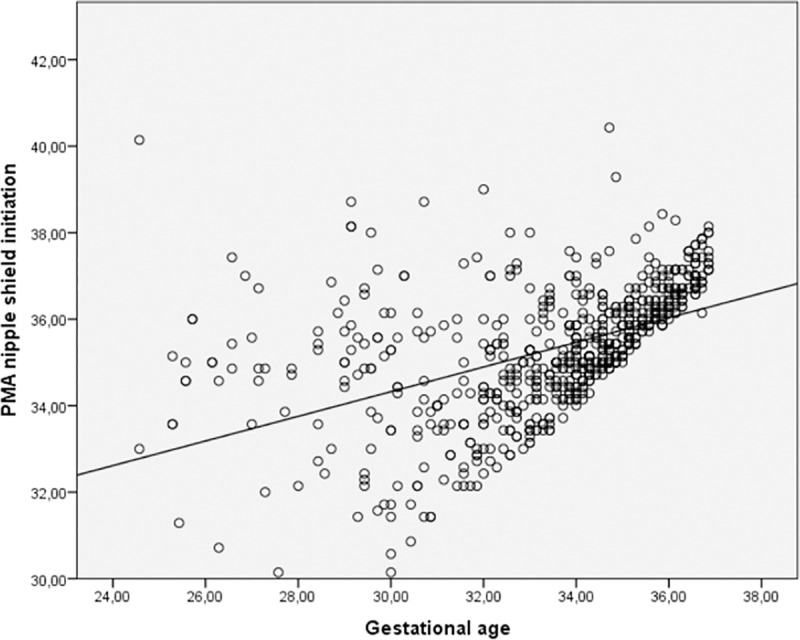
Initiation of nipple shield use according to gestational age. (R^2^ Linear = 0.239).

**Fig 3:**
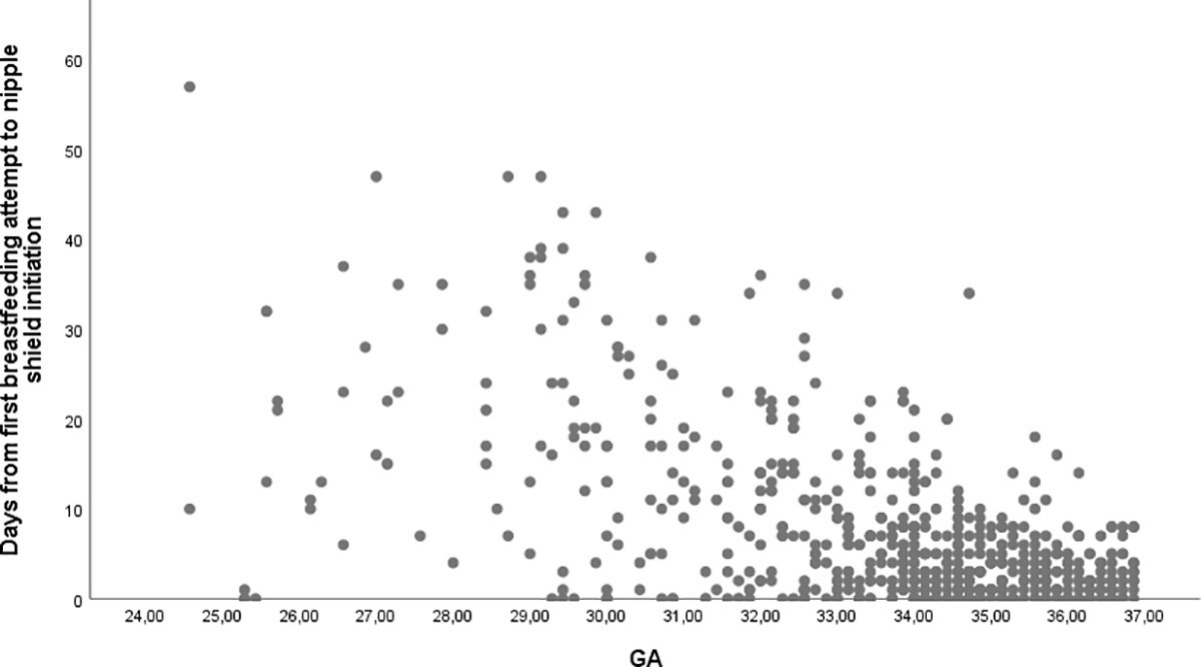
Days from first breastfeeding attempt to nipple shield initiation according to gestational age. BF = breastfeeding.

Nipple shield use was initiated at mean 35.3 weeks postmenstrual age. A moderate association between GA at birth and postmenstrual age at initiation of nipple shield use (R^2^ Linear = 0.239, p<0.0001) indicated that 24% of the variance in later initiation of nipple shield use could be explained by higher GA (Fig 2). Nipple shield use was initiated a median of 4 days after the first breastfeeding attempt (IQR 1–10 days), 17% of the infants initiated nipple shield use on the same day as the first breastfeeding attempt (Fig 3).

Bottle-feeding was introduced significantly more often in infants who used nipple shields (41%) than those who did not use nipple shields (27%) (p<0.0001). Of the 309 infants introduced to both a nipple shield and bottle-feeding, 40 (13%) were introduced to bottle-feeding before a nipple shield.

### Motives for nipple shield use

Mothers’ self-reported motives for nipple shield use appear on Table 2. The use of a nipple shield was, according to mothers, more associated with breastfeeding problems related to the infant (57%) than related to the mother (19%), and some mothers reported problems in both as motive for nipple shield use (24%). The lower the GA, the more frequently mothers reported the motives for nipple shields to be related to breastfeeding problems in the infant, and the higher GA, the more nipple shields use was reported to be related to breastfeeding problems in the mother (Table 2). Inverted or flat nipples, and breast engorgement as reported motives for nipple shield use were less common for mothers breastfeeding a preterm infant born at lower GA. Sore nipples was not a major motive for nipple shield use in mothers of preterm infants (Table 2).

**Table 2:** Motives for nipple shield use by gestational age groups.

	N*	24–27 weeks	28–31 weeks	32–34 weeks	35–36 weeks	Total**	Linear-by-Linear Ass.
		%	%	%	%	%	p-value
*Used for motives related to the infant*							
Infant slipped the nipple	708	65	63	49	48	52	0.004
Infant could not open mouth high enough to latch on	708	32	30	34	27	31	0.417
Infant became frustrated at the breast	708	36	29	24	23	25	0.092
Infant fell asleep at the breast	708	23	8	15	20	16	0.056
*Used for motives related to the mother*							
Inverted/flat nipples	604	15	14	23	30	23	0.002
Breast too engorged	604	4	2	14	22	14	<0.0001
Sore nipples	604	12	6	11	11	10	0.381

*Analysis included 708 infants and 604 mothers, respectively, who reported a motive for nipple shield use. **A nipple shield could be used for more than one motive.

### Association between nipple shield use and exclusive breastfeeding

Adjusted analyses including only those using nipple shields showed that the mothers’ self-reported motive “infant fell asleep at the breast” was associated with higher risk of not breastfeeding exclusively at discharge (OR (95% CI) 1.90 (1.15; 3.13), p = .012), and the motive “breast too engorged” was associated with lower risk of not breastfeeding exclusively at discharge (OR (95% CI) 0.32 (0.16; 0.62, p = 0.001) (Table 3). All other motives had no additional significant association with exclusive breastfeeding in this cohort.

**Table 3:** Odds for failure of exclusive breastfeeding at discharge from NICU associated with mothers’ self-reported motives for nipple shield use in preterm infants.

Adjusted analysis (N = 574)		
	OR (95% CI)	p-value
Infant slipped the nipple	0.73 (0.48; 1.10)	0.127
Infant could not open mouth high enough to latch on	0.71 (0.45; 1.11)	0.131
Infant became frustrated at the breast	1.20 (0.76; 1.90)	0.430
Infant fell asleep at the breast	1.90 (1.15; 3.13)	0.012
Inverted/flat nipples	0.94 (0.56; 1.56)	0.798
Breast too engorged	0.32 (0.16; 0.63)	0.001
Sore nipples	1.44 (0.77; 2.72)	0.257

Analysed with one infant per mother. Adjusted for gestational age (<32 weeks), multiples, gender, mode of delivery, maternal breastfeeding experience (<4 months exclusive), pumping at discharge, smoking, and site of discharge (NICU)

Significantly more of the infants who were partially or not breastfed at discharge had used nipple shields compared to infants exclusively breastfed (p< 0.0001) (Table 4). Among the 1207 infants that were exclusively or partially breastfed at discharge, significantly fewer of those who used nipple shield at discharge were exclusively breastfed at one month corrected age (50%) than those who discontinued nipple shield use before discharge (54%), and those who had never used nipple shield (58%) (p = 0.014).

**Table 4:** Breastfeeding status at discharge to home and nipple shield use during hospital stay.

	Breastfeeding status		Nipple shield use within groups	
	n/N	%	n/N	%
Exclusively breastfed	961/1406	68	469/961	49
Partially breastfed	246/1406	18	170/246	69
Not breastfed	199/1406	14	125/199	63

### Differences among NICUs

When looking at each of the 21 participating wards, the prevalence of nipple shield use by ward differed significantly between 35% and 67% of the preterm infants (p<0.001). Among the NICUs there were significant differences between mothers’ self-reported motives for introducing a nipple shield: infant slipped the nipple (<0.0001), infant could not open mouth wide enough to latch on (p = 0.007), inverted/flat nipples (p = 0.012), and breast too engorged (p = 0.017). There was a just significant moderate correlation between NICUs with higher prevalence of nipple shield use and lower rates of exclusive breastfeeding (Spearman’s R^2^ 0.201, p = 0.047) interpreted as 20% of the variance in the NICUs’ exclusive breastfeeding rates could be explained by the NICUs’ nipple shield rate.

## Discussion

We found a high prevalence in the use of nipple shields and a huge variation in the time from first breastfeeding attempt to initiation of nipple shield use in the large cohort of preterm infants. We have described in detail the distribution of mothers’ self-reported motives for nipple shield use, and how these motives were more related to breastfeeding problems in infants than in mothers. In the present analyses we found an association between the motive for nipple shield use “Infant fell asleep at the breast” and failure of exclusive breastfeeding at discharge.

### Strengths and limitations

The strengths of the present study are the large number of participants including preterm infants from 24 to 36 gestational weeks, the national multicenter design including all neonatal wards that care for preterm infants during breastfeeding establishment, the level of specificity among users of nipple shields, and defining breastfeeding as exclusive breastfeeding at and from the breast.

The design of the cohort study of nipple shield use had the limitation that infants who were exposed to nipple shields also may be those who had more breastfeeding problems that could complicate breastfeeding establishment regardless of nipple shield use. This observational study was not designed to establish cause and effect relationships but instead associations. The huge variation in nipple shield use between the participating NICUs (35–67% of discharged preterm infants) indicated that nipple shields were not always used to solve the same degree of breastfeeding problems. Nevertheless, a large study like this provides evidence from clinical practice that adds to results from smaller studies. The results should be interpreted with caution as many of the mother-infant dyads had more than one motive for nipple shield use and it is unclear whether mothers or staff initiated nipple shield use.

### Prevalence and characteristics of nipple shield users

The present study had a high prevalence of nipple shield use (54%). More restrictive use has been found in a study from a NICU in the UK with only 2% using nipple shields [1]. Another Danish cohort including 363 preterm infants also found relatively high prevalence (41%) among the preterm infants [3]. To our knowledge, no further rates of nipple shield use in preterm infants have been published. The high prevalence in Denmark could be explained by the Danish Health Board recommending nipple shield use for preterm infants with reference to a US study from 2000 [5]. For some mothers, the use of a nipple shield may rescue breastfeeding when the infant would otherwise have been unable to breastfeed [3], but the present prevalence of 54% in preterm infants seems too high a rate for nipple shield use. The characteristics of nipple shield users are similar to findings in a study of term and preterm mother-infant dyads [3] and indicates that mothers with less experience in breastfeeding are more likely to use a nipple shield.

### Motives for nipple shield use

Nipple shields were, according to the mothers, used for many different motives in the present study. The motive “infant slipped the nipple” seems to be the most common motive for nipple shield use in preterm infants, as this was also found in a US study (52% in the present study and 63% in the US study [5]). Using nipple shield for the motive “infant fell asleep at the breast” was less common in this Danish cohort than in the US study (13% compared to 29% [5]).“Accustomed to a firmer teat” was not a motive for nipple shield use in the present study compared to nearly half of the mothers in the UK study [1]. This may be because the standard of care in Danish NICUs is to avoid bottle-feeding except when exclusive breastfeeding is no longer the mother’s goal, which is in line with the Neo-BFHI recommendations [11, 22]. Of the infants in the present study who were introduced to both nipple shield and bottle-feeding before discharge, 13% were introduced to bottle-feeding before nipple shield or on the same day. The other studies do not clearly state how many of the preterm infants were exposed to bottle-feeding before nipple shield use was initiated. The differences in motives between studies of nipple shield use could be due to different sample sizes or different practices: whether nipple shield use was restrictive, and whether bottle-feeding was introduced before a nipple shield.

The motives for nipple shield use related to gestational age groups in preterm infants are unique, as this has not been previously demonstrated. The motive “infant slipped the nipple” was not only the most frequent motive but was also a larger problem for infants with lower GA. The two motives for nipple shield use, “flat/inverted nipples” and “breast too engorged”, that related to the mother were significantly less frequent among mothers of infants with lower GA. Infants with lower GA established breastfeeding at a higher postnatal age and after lactogenesis stage II [8] and the mother’s breast may therefore be softer and easier to latch on. The reason may also be that breastmilk pumping before breastfeeding establishment—maybe for many weeks—could shape the nipple and make it easier for the infant to latch.

### Association between nipple shield use and exclusive breastfeeding

The motive for nipple shield use “breast too engorged” could be regarded as save for establishment of exclusive breastfeeding, as we found a low OR for failure of exclusive breastfeeding. The motive for nipple shield use “infant fell asleep at the breast” was associated with not breastfeeding exclusively at discharge. The multiple regression analysis was performed only with mothers who had used a nipple shield and therefore from the beginning had higher risk of not establishing exclusive breastfeeding as a former analysis of this cohort showed that use of a nipple shield was associated with a more than double OR for not breastfeeding the preterm infant exclusively at discharge (OR 2.3 (95% CI 1.6; 3.2), p<0.0001) [2].Even though studies have described that some mothers find the use of a nipple shield to be helpful [3, 23, 24] and 49% of the exclusively breastfed infants in our study had used a nipple shield, it appears from the present study, that using a nipple shield did not solve the problem for several infants. We found 69% of those establishing partially breastfeeding and 63% of those not establishing breastfeeding had used nipple shields. More of the motives for nipple shield use described immature sucking skills (infant slipped the nipple, could not open mouth high enough, and fell asleep), and not a persistent breastfeeding problem, and we found no positive association between these motives for nipple shield use and exclusive breastfeeding at discharge. Nipple shield use has previously been recommended to infants falling asleep at the breast [5], which is contrary to our findings. The contrary results could be because the US study, upon which the wide recommendation of nipple shields to preterm infants is built, included only 34 infants, had no direct comparison group, and was supported by a company producing nipple shields. Also, the US study defined breastfeeding as “any breastmilk feeding”, which could be the reason for the different interpretations of how nipple shield use is associated with breastfeeding duration. It is likely that exclusive breastfeeding [2, 3, 7] could be more influenced than any breastmilk-feeding [5] by nipple shield use. Defining breastfeeding as breastmilk feeding in such studies could detect mothers’ ability to pump breastmilk and not the infant’s ability to continue sucking at the breast and extract milk from the breastwhy duration of breastmilk feeding should not be used in arguing for the safety of nipple shield use when exclusive breastfeeding at the breast is the goal. Even though Meier and Clum found that preterm infants consumed more breastmilk with the nipple shield [4, 5], our results indicate that nipple shield use is not facilitating nor accelerating exclusive breastfeeding.

### Differences among NICUs

We found significant differences in nipple shield use between the participating NICUs both in prevalence and in motives for nipple shield use, demonstrating that it is possible to reduce the use of nipple shields Nipple shield use could be regarded as an indicator for breastfeeding problems [3] and is often suggested by the staff in the NICU rather than the mother [23], that means the culture and standard of care in the NICU might decide whether nipple shields are used as first choice or last choice to solve breastfeeding problems and whether nipple shields are only used for very good reason to solve major breastfeeding problems or regarded as a helpful device also for minor problems [23]. We found a correlation between NICUs with high rates of nipple shield use and low rates of exclusive breastfeeding. If use of nipple shields would be positively associated with breastfeeding, we would have expected that NICUs with high rates of nipple shield use would have higher or same rates of exclusive breastfeeding as NICUs with more restrictive nipple shield use. Implementing the evidence-based recommendations for nipple shield use outlined in the Neo-BFHI [11] could minimize the variations in clinical practice. We did find that nipple shield use was initiated very early (from 30 weeks PMA) in some infants or very close to the first breastfeeding attempt. Staff impatience has previously been described as a motive for nipple shield use [23]. Especially for the smallest infants, the early initiation of nipple shield use did not reflect patience. Former analyses of this cohort found that exclusive breastfeeding was not established earlier in mother-infant dyads using a nipple shield compared to those not using nipple shield (p = 0.743) [8], so it is likely that nipple shield use will not help staff impatience but instead might put the mother-infant dyad at risk of not establishing exclusive breastfeeding. We therefore suggest accepting the infant’s inability to breastfeed larger amounts of milk and being patient, i.e. allowing the infant just to lick, taste, and suck a little while waiting for the infant to develop the necessary breastfeeding skills supported by prolonged skin-to-skin contact [8].

## Conclusion

We found a high prevalence of nipple shield use (54%) in preterm mother-infant dyads and a huge variation in the time from first breastfeeding attempt to initiation of nipple shield use in the large cohort of preterm infants. Motives for nipple shield use were more related to breastfeeding problems in infants than in mothers, and there were differences between GA-groups in motives for nipple shield use. We found an association between the motive for nipple shield use “Infant fell asleep at the breast” and failure of exclusive breastfeeding at discharge. The present study does not give justifiable reasons for nipple shield use, except for “breast too engorged”. Nipple shields should not be recommended for infants falling asleep at the breast. Instead, staff and mothers should be patient, allowing the dyad time skin-to-skin. Mothers of preterm infants should be given skilled breastfeeding support to solve breastfeeding problems and should repeat attempts to breastfeed without a nipple shield. The present study indicates that the use of a nipple shield does not promote exclusive breastfeeding in preterm infants. Therefore, the results of initiation time and the distribution of motives should not be used as recommendations for nipple shield use. Nipple shield use should be restricted and at the lowest possible rate.
